# Alpha-defensins 1-3 release by dendritic cells is reduced by estrogen

**DOI:** 10.1186/1477-7827-9-118

**Published:** 2011-08-23

**Authors:** Maria M Escribese, Marta Rodríguez-García, Rhoda Sperling, Stephanie M Engel, Teresa Gallart, Thomas M Moran

**Affiliations:** 1 Department of Microbiology, Mount Sinai School of Medicine, New York, NY, USA; 2 Immunology Institute, Mount Sinai School of Medicine, New York, NY, USA; 3 Department of Molecular Microbiology and Infections Biology, CIB, CSIC, Madrid, Spain; 4 Service of Immunology, Hospital Clinic Universitari de Barcelona, Barcelona, Spain; 5 Department of Physiology, Dartmouth Medical School, Lebanon, New Hampshire, USA; 6 Department of Obstetrics, Gynecology and Reproductive Sciences, Mount Sinai School, of Medicine, New York, NY,USA; 7 Department of Preventive Medicine, Mount Sinai School of Medicine, New York, NY, USA

## Abstract

**Background:**

During pregnancy the immune system of the mother must protect any activation that may negatively affect the fetus. Changes in susceptibility to infection as well as resolution of some autoimmune disorders represent empirical evidence for pregnancy related alterations in immunity. Sex hormones reach extremely high levels during pregnancy and have been shown to have direct effects on many immune functions including the antiviral response of dendritic cells. Among the immunologically active proteins secreted by monocyte derived DCs (MDDC) are the alpha-defensins 1-3. This family of cationic antimicrobial peptides has a broad spectrum of microbicidal activity and has also been shown to link innate to adaptive immunity by attracting T cells and immature DCs, which are essential for initiating and polarizing the immune response.

**Methods:**

We compare culture-generated monocyte derived DCs (MDDCs) with directly isolated myeloid dendritic cells (mDCs) and plasmacytoid dendritic cells (pDCs) and measure their alpha-defensins 1-3 secretion by ELISA both, in basal situations and after hormone (E2 or PG) treatments. Moreover, using a cohort of pregnant women we isolated mDCs from blood and also measure the levels of these anti-microbial peptides along pregnancy.

**Results:**

We show that mDCs and pDCs constitutively produce alpha-defensins 1-3 and at much higher levels than MDDCs. Alpha-defensins 1-3 production from mDCs and MDDCs but not pDCs is inhibited by E2. PG does not affect alpha-defensins 1-3 in any of the populations. Moreover, alpha-defensins 1-3 production by mDCs was reduced in the later stages of pregnancy in 40% of the patients.

**Conclusions:**

Here, we demonstrate that mDCs and pDCs secrete alpha-defensins 1-3 and present a novel effect of E2 on the secretion of alpha-defensins 1-3 by dendritic cells.

## Background

Defensins are a family of cationic antimicrobial peptides [1] that demonstrate a broad-spectrum of microbicidal activity against bacteria, fungi and viruses [2]. Moreover, recent evidence suggest that defensins also interact with host immune cells playing an important role in both the innate and adaptive immune response [3]. Two groups of human defensins have been described based on their structural characteristics: α- and β-defensins [1]. Both types have antimicrobial activity against bacteria and fungi as well as against enveloped virus such as influenza, herpes virus, cytomegalovirus and HIV [2]. α-defensins 1-3 specifically, are secreted by monocytes, monocyte-derived DCs [4], NK cells and γδT cells in addition to neutrophils [2]. Moreover, α-defensins 1-3 have been detected throughout the female reproductive tract (FRT) in non-pregnant women [5,6] as well as in the amnion, chorion, placenta, amniotic fluids and cervical mucus plug from pregnant women [7-9]. Changes in these peptides have also been reported to be involved in several pregnancy complications such as preterm delivery and preeclampsia [7,10].

Dendritic cells (DCs), as immunological sentinels, are crucial intermediates linking innate immunity to adaptive immunity. Upon exposure to microbial invaders, they undergo a maturational change characterized by the up-regulation of surface molecules involved in the interaction with T cells and the release of numerous cytokines. This culminates in their migration to lymph nodes, where microbe-derived peptides are presented to specific T cells and initiation of the adaptive immune response occurs [11,12]. Most studies utilize DCs generated from CD14^+ ^monocyte precursors, monocyte derived dendritic cells (MDDCs) cultured in cytokine-supplemented media. However, fully functional DCs can be directly isolated from human blood based upon the surface expression of CD1c^+ ^(mDCs) without the need for cytokine differentiation. Other DC populations such as the plasmacytoid DCs (pDCs) have been proposed as important mediators of innate immunity because of their ability to produce large amounts of type I interferon (IFN) following viral infection.

Steroid sex hormones exert a powerful effect on various immune system functions [13,14]. The monthly hormonal changes associated with the female menstrual cycle also correlate with deviations in immunity [15]. However, the most drastic changes in immunity occur during pregnancy when the levels of estrogen (E2) and progesterone (PG) rise to extremely high concentrations in the third trimester [16,17]. Some defensins, such as HD-5 and HBD1-4 undergo variations at mucosal surfaces during the menstrual cycle [6,18], suggesting that α-defensins 1-3 may be regulated by changes in hormonal levels [19].

Numerous interactions between the endocrine and the immune systems have been described [20-22]. Several groups have shown that estrogen administration leads to clinical improvement in experimental autoimmune encephalomyelitis due to changes in DCs function and the promotion of a Th2 immune response [23]. In contrast, disease exacerbation has been reported to occur during pregnancy in systemic lupus erythematosus (SLE) patients [24]. Progesterone (PG) also causes a shift to a Th2 dominated response by blocking the Th1 pathway, inducing IL-10 and blocking IL12 [13,14].

E2 has a wide range of physiological activities, and it is thought to act through two receptors ERα and ERβ by direct and indirect binding to promoter and/or enhancer regions within target genes [25]. Receptors for PG and E2 have been detected on some leukocytes [26,27] and in particular on DCs [28]. At least three distinct classes of anti-estrogens have been described. In this study we have used a type I antagonists represented by ICI 182,780 that inhibits estrogen activity both *in vivo *and *in vitro*. In contrast, type II and III, represented by Tamoxifen and Raloxifene mimic the biological effects of E2 in some specific tissues but oppose E2 action in other [29].

In this report we demonstrate that directly isolated DCs secrete α-defensins 1-3, and that E2 acts to inhibit this secretion. This same trend is observed in mDCs isolated from pregnant women in their first trimester (low E2) and third trimester (high E2). Together these data suggest a direct effect of E2 over the secretion of α-defensins 1-3 by dendritic cells.

## Methods

### Isolation and culture of human DCs

PBMCs were isolated as previously described [30]. Briefly, Ficoll density gradient centrifugation (Histopaque; Sigma Aldrich) was performed using buffy coats from healthy human donors (Mount Sinai Blood Donor Center and New York Blood Center). CD14^+ ^cells were immunomagnetically purified using anti-human CD14^+ ^antibody-labeled magnetic beads and iron-based Midimacs LS columns (Miltenyi Biotec). After elution from the columns, these cells were plated (0.7 × 10^6 ^cells/ml) in DC medium (RPMI [Invitrogen], 1% Human Serum, 100 units/ml of penicillin, and 100 μg/ml streptomycin [Invitrogen]) supplemented with 500 U/ml human granulocyte-macrophage colony-stimulating factor (GM-CSF; Peprotech) and 1,000 U/ml human interleukin-4 (IL-4; Peprotech) and incubated for 5 to 6 days at 37°C. mDC and pDCs were immunologicaly purified using anti-human CD1c (BDCA-1) and CD303 (BDCA-4) antibody-labeled magnetic beads (Miltenyi Biotec) and iron based MiniMacs LS columns. After elution from columns, cells were plated (1 × 10^6 ^cells/ml) and treated or not with E2 or PG.

### Hormone treatment and viral infection

After 5 to 6 days in culture at a 1 × 10^6 ^cell/ml concentration, MDDCs were treated either with 17β-estradiol (E2; Sigma) or with progesterone (PG; Sigma) at concentrations of 10 μg/ml, 1 μg/ml, 0.1 μg/ml and mock (0 ug/ml). In some cases the MDDCs treated for 24 h with 10 ug/ml of E2 or PG were infected with New Castle Disease Virus NDV-B1 for 10 h at and MOI **(**multiplicity of infection) of 0.5. mDCs and pDCs were treated with each specific condition right after their isolation. After mock or E2/PG treatment supernatants were collected and α-defensins 1-3 secretion was measured by ELISA.

### ICI 182,780 treatment

MDDCs at day 2 in the differentiation process in a concentration of 1 × 10^6 ^cell/ml are treated with 10, 100 and 1000 nM of either ICI 182,780 (AstraZeneca) or the vehicle (DMSO) for 3 days until day 5 of the differentiation process. mDCs were pretreated with ICI 182,780 for 24 h previous to the hormonal treatment. Cell viability, in both cell populations, was measured before and after treatment by Trypan Blue (Invitrogen) exclusion. After ICI 182,780 pre-treatment DCs were either mock or E2 treated for 24 h and supernatants and cell lysates were collected for further analysis.

### ELISAs

Capture enzyme-linked immunosorbent assays (ELISAs) for HNP1-3 (Hycult Biotechnology) was used according to the manufacturers' instructions to quantify the α-defensins 1-3 in the DC supernatants from pregnant women. Plates were read in an ELISA reader from Biotek Instruments assay following the manufacturer's protocol and data were analyzed using software from Applied Cytometry Systems. Levels of serum estradiol and progesterone were measured by Lenetix Medical Screening Laboratory, Inc., Mineola, New York.

### Flow Cytometry

Cells were stained with CD14 FITC/CD11cPE (from BD Bioscience), to verify the differentiation process from monocytes to MDDCs. The staining was performed according to the manufacturer's instructions (Beckman Coulter or BD-Pharmingen). The expression level of each marker was determined by flow cytometry after gating on the lineage-specific marker, using a FC 500 flow cytometer from Beckman Coulter. Data were analyzed using CXP software (Beckman Coulter).

### RNA extraction and Quantitative real-time PCR from human DCs

RNA extraction and RT-PCR was performed as described before [30]. Briefly, samples of 1 × 10^6 ^DCs differentially treated according to the experimental protocol were pelleted, and RNA was isolated and treated with DNase using Absolutely RNA RT-PCR miniprep kit (Stratagene, La Jolla, CA, USA). RNA was quantified using a Nanodrop spectrophotometer (Nanodrop Technologies). The yields of RNA were approximately 20-50 ug/sample. qRT-PCR was performed by using a previously published SYBR green protocol with an ABI7900 HT thermal cycler [30]. Each transcript in each sample was assayed two times, and the mean cycle threshold was used to calculate the fold change for each gene. Four housekeeping genes were used for global normalization in each experiment (actin, Rps11, glyceraldehyde-3-phosphate dehydrogenase, and tubulin genes) [31]. Data validity by modeling of reaction efficiencies and analysis of measurement precision was determined as described previously [30].

### Patient samples

Maternal blood and serum samples utilized in these experiments were obtained from the *Viral Immunity in Pregnancy (VIP) *study, an ongoing NIH-funded project which enrolls pregnant women during the first 20 weeks of gestation and follows them through 6 months post delivery in order to assess the interplay between maternal systemic immunologic responses and enhanced susceptibility to viral pathogens. The study is IRB approved by the Program for the Protection of Human Subjects at the Mount Sinai School of Medicine. Peripheral blood samples are collected from women at five specific time points: study entry (< 20 weeks' gestation), 25-27 weeks' gestation, 34-36 weeks' gestation, 6 weeks post-delivery and 6 months post-delivery. Each woman's perinatal or 6 weeks postpartum blood draw can be compared against her own 6 month post-partum "normal" blood draw, thus allowing each woman to serve as her own control, implicitly accounting for interindividual variation in a number of factors. Women are recruited from the general obstetrics practice at the Mount Sinai Hospital in NYC. Exclusion criteria include: (1) Individuals with known or suspected HIV infection; (2) Individuals with a history of splenectomy, known auto-immune disorders (systemic lupus erythematosus or rheumatoid arthritis), or require chronic systemic immunosuppressive therapy; (3) Individuals with a history of previous bone marrow or organ transplant; (4) Individuals who have received any immune globulin (including Rhogam) or blood derived products within 3 months of study enrollment; (5) Individuals who became pregnant using a donor egg for this pregnancy; (6) Individuals with a history of malignancy; and, (7) Individuals that will be managed in the third trimester of pregnancy with epogen. Individuals with asthma were eligible to participate as long as they did not receive chronic oral steroid treatment. Individuals with anemia, defined by a Hemoglobin < 10 g/dl were excluded by the local IRB because of safety concerns re: volume of blood donation required by the study.

### Statistical Analysis

Data were transformed with natural-logarithms. Mixed models were used to examine changes in defensins during pregnancy accounting for repeated measures taken from the same women over time. The analyses were performed using PROC MIXED of SAS, Version 9.2 (SAS Inc., Cary, NC). All P values were two-tailed, with P < 0.05 considered as statistically significant. Statistical analysis for changes in a-defensins 1-3 induce by hormones treatments was performed by analysis of variance, and a *P *value less than .05 was considered significant.

## Results

### Myeloid DCs (mDCs) and plasmacytoid (pDCs) secrete a-defensins 1-3

**Figure 1:**
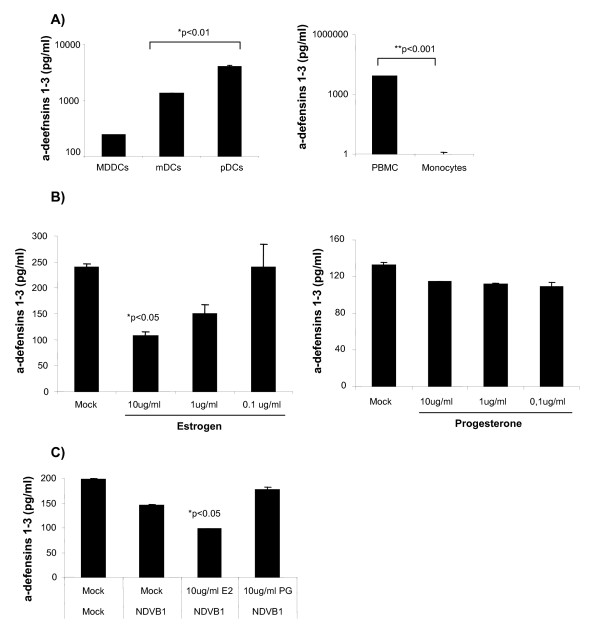
**MDDCs secrete α-defensins 1-3: E2 and PG effect**. A) ELISA quantification of *a*-defensins 1-3 secreted by MDDCs, mDCs and pDCs (left panel) or PBMCs and monocytes (right panel) in the supernatant after 6 h of culture. Data are presented as mean plus or minus SD from 3 independent experiments.*P < 0.01 and *P < 0.001 versus MDDCs levels. Data are presented in a log scale. Levels of α-defensins 1-3 measured by ELISA in: (B) MDDCs treated for 24 h with 10, 1 and 0.1 ug/ml of E2 (left panel) or PG (right panel). (C) MDDCs pretreated for 24 h with E2 or PG (10 ug/ml) before NDV-B1 infection for 10 h. Data are presented as mean plus or minus SD from 3 independent experiments. *P < 0.05versus MDDCs levels.

We have previously reported that α-defensins 1-3 levels were almost undetectable in monocytes but when differentiated into DCs (MDDCs) after 7 days in culture, they produce and secrete α-defensins 1-3 [4]. In order to determine if these cultured cells were representative of DC populations circulating in vivo, mDCs (CD1c+) and pDCs (BCDA-4+) were directly isolated from buffy coats as well as MMDCs. After 6 h in culture the levels of α-defensins 1-3 secreted into the supernatants were measured. As a control we used monocytes (negative control) and PBMC (positive control), that has been described to secrete α-defensins 1-3 constitutively [32] (Figure 1A, right panel). A comparison of the constitutive secretion of α-defensins 1-3 from MDDCs, mDCs and pDCs by ELISA show that the levels of α-defensins 1-3 detected in the supernatants from mDCs and pDCs were 10 and 15-fold higher, respectively, than those detected in the supernatants from MDDCs (Figure 1A, left panel) (p < 0.01).

### Estrogen but not Progesterone down-regulates a-defensins 1-3 secretion by DCs

Estrogen exerts an inhibitory effect on mDC and MDDC secretion of type I interferon (IFN) and some inflammatory cytokines [28]. Therefore we measured the effect of estrogen (E2) and progesterone (PG) on α-defensins 1-3 secretion by MDDCs, mDCs and pDCs. Treatment of MDDCs with E2 resulted in a dose-dependent reduction in α-defensin 1-3 secretion. Conversely, PG treatment had no effect on the secretion of α-defensins 1-3 in MDDCs (Figure 1B). Moreover, we also show that E2 but not PG still have a significant (*p < 0.05 vs mock treated DCs) inhibitory effect in the secretion of α-defensins 1-3 in viral infected MDDCs (Figure 1C), that correlates with our previous report describing the immunosuppressive effect of E2 during viral infection [28].

**Figure 2:**
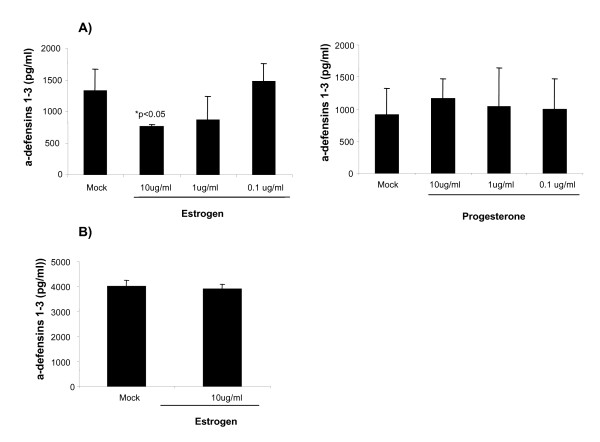
**mDCs and pDCs secrete α-defensins 1-3: E2 and PG effect**. Levels of α-defensins 1-3 measured by ELISA in: (A) mDCs treated for 24 h with 10, 1 and 0.1 ug/ml of E2 (left panel) or PG (right panel). (B) pDCs treated with 10 ug/ml of E2 for 8 h. Data are presented as mean plus or minus SD from 3 independent experiments.*P < 0.05 versus Mock treated DCs.

We also analyzed the effect of E2-treatment on the secretion of α-defensins 1-3 by mDCs and in accordance with the results observed for MDDCs, a significant reduction in the levels of α-defensins 1-3 was detected in cells treated with high concentrations of E2 but PG treatment did not have any significant effect (Figure 2A). In contrast, the α-defensins 1-3 secretion by pDCs was not affected by estrogen (Figure 2B). These results show that E2 suppresses α-defensins 1-3 secretion by mDCs and MDDCs but not in pDCs.

### Effect of ICI 182,780 in the secretion of α-defensins 1-3 by DCs

**Figure 3:**
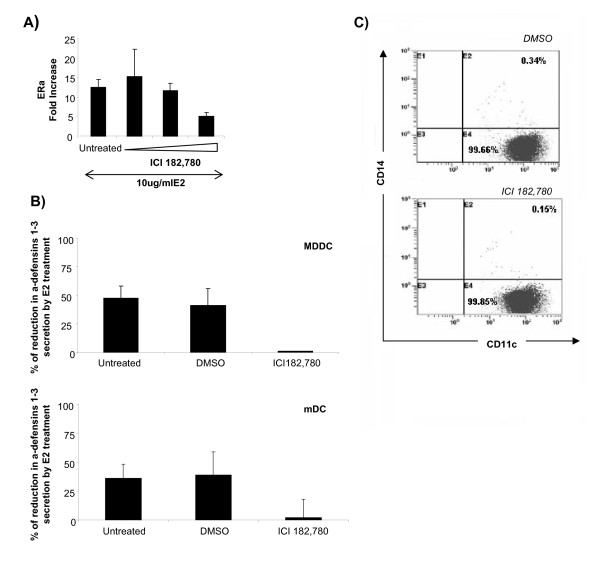
**ICI 182,780 revert E2-inhibitory effect in the release of a-defensins 1-3 by DCs**. CD14^+ ^cells isolated from fresh blood were cultured for 5 days in the presence of IL-4 and GM-CSF to differentiate into DCs. The cells were treated with 10, 100 and 1000 nM of ICI 182,780 for the last 3 days of the differentiation process. (A) Levels of ERa measured by RT-PCR in DCs treated or not with ICI 182,780 in the presence of E2 (10 ug/ml) for 24 h. The data presented as mean ± SD are representative of three independent experiments. (B) Percentage of reduction induces by E2-treatment in the levels of a-defensins 1-3 secreted by MDDCs and mDCs pre-treated or not for 3 days (MDDCs) or 24 h (mDCs) with 1000 nM of ICI 182,780 or DMSO, prior to 24 h of Estrogen or Mock treatment.. The data presented as mean ± SD are representative of three independent experiments. (C) Percentage of CD11c+CD14+ cells measured by FACS after 5 days of differentiation in the presence of ICI 182,780 or DMSO during the last 3 days of the process. These data are representative of three independent experiments.

In order to determine if the reduction of α-defensins 1-3 secretion was specific and mediated through the estrogen receptor, DCs were treated with the estrogen receptor (ER) antagonist ICI 182,780 at a concentration of 1000 nM, 100 nM and 10 nM for 3 days prior to the E2 treatment. Since E2 upregulates ER expression, ERα transcription in the presence of E2 is a useful measure of the effectiveness of ICI 182,780. At the highest concentration of ICI 182,780 in the presence of 10 ug/ml E2 a significant reduction of 2.5 -folds in ERα expression was observed (Figure 3A). This concentration of the drug was able to reverse the inhibition of E2 on α-defensins 1-3 secretion by both types of DCs (Figure 3B). However, low concentrations for Faslodex (100 and 10 nM) do not significantly change (1.24 and 0.92- folds, respectively) ERα expression in relation with the levels detected in untreated MDDCs. Moreover, 1000 nM ICI 182,780 did not affect the differentiation process of the MDDCs measured by CD11c and CD14 expression at day 5 of culture (Figure 3C). These results confirmed that the effect of E2 on α-defensins 1-3 secretions was mediated through estrogen receptor signaling and that a novel E2 regulated mechanism prevents α-defensins 1-3 secretion in DCs.

### Secretion of a-defensins 1-3 by DCs during pregnancy

**Figure 4:**
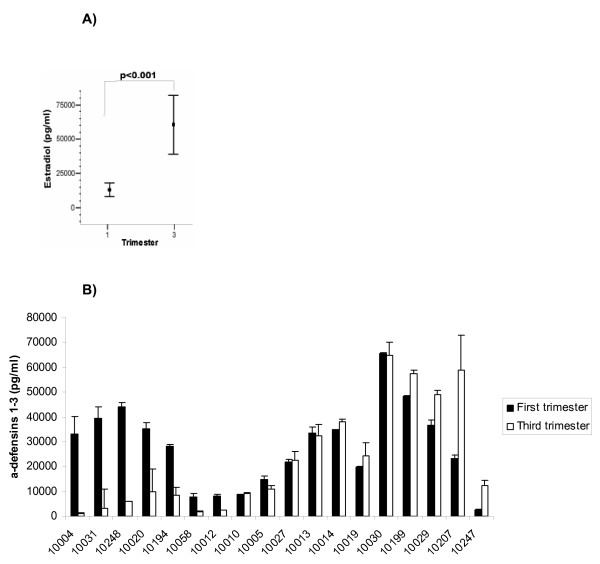
**Release of a-defensins 1-3 by mDC during pregnancy**. (A) Levels of Estradiol measured by ELISA in serum samples from pregnant women (n = 18) during first and third trimester. *p < 0.001 vs first trimester. (B) Levels of a-defensins 1-3 secreted by mDC from all the individual patients (n = 18) at first and third trimester. The data are presented as mean ± SD.

Since estrogen levels rise significantly in women from the first to the third trimester of pregnancy we speculated that if estrogen was exerting regulatory control over α-defensins 1-3 secretion, then the mDCs collected late in pregnancy should produce less of these anti-microbial peptides. A cohort of women from an ongoing study of immunity during pregnancy being performed at Mount Sinai School of Medicine (VIP study) were chosen based upon their significant difference in serum estrogen levels between the first and third trimester (Figure 4A). mDCs were isolated from these subjects and the secretion of α-defensins 1-3 was measured by capture ELISA after 12 h in culture. However, patient variation was great and no statistical difference between the groups could be demonstrated. α-defensins 1-3 release by DCs isolated from 40% of the patients showed at least a two-fold reduction between the 1^st ^and 3^rd ^trimester as estrogen levels rose. However, many (50%) showed no significant change and two patients showed a more than a two fold rise in secretion (10%) (Figure 4B).

## Discussion

During normal pregnancy, the maternal immune system remains systemically immunocompetent [33,34]. However, it is faced with a dilemma that it must continue to protect the mother against infection while inhibiting immune activation against the semiallogeneic fetus. There is clear evidence that in the later stages of pregnancy women are uniquely susceptible to microbial infection. The prevailing view, though not shared by all, is that the predominant immune response is shifted from a gamma interferon based Th1 response to a less inflammatory Th2 response. The period of enhanced sensitivity to infection corresponds to the extremely high concentrations of E2 and PG that are reached in the later stages of pregnancy [16,17]. Both E2 and PG have been shown to exert a powerful effect on various aspects of the innate and adaptive immune response [13,14]. In a previous publication we investigated the effect of these hormones on the activation of DCs cultured from monocytes as well as those isolated directly from the blood. PG did not exert any effect on DCs in either the steady state or after activation by viruses. In contrast, E2 was a potent inhibitor of DC activation triggered by a number of human viruses but not by toll-like receptors ligands (TLRs) [28].

Alpha-defensins 1-3 are small peptides that can act both as natural antibiotics by directly killing a wide range of microorganisms and by participating in adaptive immunity by attracting monocytes, T cells, or immature DCs [35,36]. Moreover, α-defensins 1-3 have been detected throughout the female reproductive tract (FRT) in non-pregnant women [5,6] as well as in the amnion, chorion, placenta, amniotic fluids and cervical mucus plug from pregnant women [7-9]. Previous reports by us and others demonstrate the expression of α-defensins 1-3 by immune cells populations including MDDCs [4], NK cell, B cells, γδTcells, mast cells, neutrophils [2]. Most human DC studies are performed using MDDCs produced by culturing these cells in the presence of the cytokines GM-CSF and IL4. It is not known whether cells produced this way simulate in vivo DC populations. However, mDCs as well as pDCs can be isolated directly from human blood by the expression of the CD1c marker (mDC) or BCDA4 (pDC) on their surface [37]. In this report we analyzed directly isolated mDCs and pDCs and show that they both secrete significantly more α-defensins 1-3 than MDDCs. These results suggest that culturing of DCs may reduce their α-defensin 1-3 secretion and that previous work may have underestimated the contribution of DCs to in vivo α-defensin 1-3 production [38].

17-β-estradiol (E2), one of the most important forms of estrogen, acts through binding to two known hormone receptors, estrogen receptors ERα and ERβ [25], which are expressed in specific tissues as well as in immune cells, including macrophages, CD8^+ ^T cells, CD4^+ ^T cells, B cells, monocytes, and DCs [39]. Cells pre-treated with ICI 182, 780, a specific ER antagonist, became insensitive to the inhibitory effect of E2, demonstrating a direct and specific effect of E2 on the release of α-defensins 1-3 by DCs. However, intrinsic or subsequently acquired resistance to the therapy remains a major obstacle in treatment with this compound as has been describe by Zhao et al., using a cell line of breast cancer [40]. These open new questions that should be addressed in further investigations with dendritic cells. In our system, ICI 182,780 reduces the expression of the ER, as a result of the induction of a proteasome-dependent degradation induced by this compound [41]. However, ICI 182,780 treatment does not affect the differentiation of monocytes into MDDCs as measured by percentage of CD11c^+^CD14^- ^cells.

E2 but not PG suppressed the α-defensins 1-3 production from the mDCs at high concentration but had no effect on pDC α-defensins 1-3 secretion. This result is consistent with our earlier observation that E2 did not inhibit activation or cytokine secretion from pDCs. A possible explanation for these observations comes from the work of Hartman et al who demonstrated that murine pDCs derive exclusively from estrogen-resistant myeloid progenitors [42] which may explain the failure of E2 to alter α-defensin 1-3 production by these cells.

Based upon our in vitro studies we expected that mDCs collected from the blood in the later part of pregnancy would show a reduction in α-defensins 1-3 production. Moreover, evidence suggests that elevated levels of α-defensins 1-3 in serum correlated with adverse pregnancy outcomes [43,44]. While we observed a trend towards less α-defensins 1-3 production from DCs collected in the 3^rd ^trimester relative to those collected from the same patient in the 1^st ^trimester, the difference was not statistically significant. Almost 40% of the patients did show a very substantial drop in α-defensins 1-3 release from their mDCs at third trimester, while the remaining patients secreted α-defensins 1-3 at nearly identical levels to what was observed in the 1^st ^trimester. It is worth noting, however, that the levels of estrogen detected in serum were lower than the dose that most effectively inhibited α-defensin 1-3 secretion in vitro.

Among the 50% failed to show a reduction, the consistency of the mDC α-defensin 1-3 secretion within patients and variation between patients (ranging from 10 ng/ml to 60 ng/ml) was significant. This high inter-donor variability has been well described and likely corresponds to variations in the copy number of the genes that encode for α-defensins 1-3 between individuals [45].

The sum of the α-defensin 1-3 production by mDCs suggested a net loss in the later stages of pregnancy. If other cells that produce α-defensins 1-3 were likewise affected, serum α-defensins1-3 during pregnancy would be expected to drop.

In this report we document that myeloid and plasmacytoid DCs directly isolated from the blood produce substantial amounts of α-defensins 1-3 constitutively. pDCs are not affected, but both mDCs and MDDCs produce less α-defensins 1-3 in the presence of high levels of E2. Alpha-defensins 1-3 release from mDCs collected in the third trimester of pregnancy either fell or stayed the same in the majority of the patients.

## Conclusions

Our results demonstrate a specific effect for E2 on the secretion of a-defensins 1-3 by dendritic cells. Moreover, we also observed this effect in dendritic cells isolated from pregnant women at third trimester, when the levels of E2 reach extremely high concentrations. Together, this data provide new insights that contribute to explain immune changes during pregnancy.

## List of abbreviations

DC: Dendritic cells; FRT: Female reproductive track; NK: Natural killer; E2: Estrogen (17-β-estradiol); PG: Progesterone; HD5: alpha-defensin HD5; HBD1-4: Human beta defensins 1-4; MDDC: Monocyte derived dendritic cells; mDCs: myeloid dendritic cells; pDCs: plasmacytoid dendritic cells; IFN: Interferon; ERα: Estrogen receptor alpha; ERβ: Estrogen receptor beta; PBMC: Peripheral blood mononuclear cells.

## Competing interests

The authors declare that they have no competing interests.

## Authors' contributions

MME designed and performed research, analyzed data, and wrote the manuscript; MGR performed research and wrote the manuscript; SE performed statistical analysis; RS wrote the manuscript; TG designed research and wrote the manuscript; and TMM designed research and wrote the manuscript. All authors read and approved the final manuscript
